# Review of the Chinese leafhopper genus *Parazyginella* Chou & Zhang (Hemiptera, Cicadellidae, Typhlocybinae, Zyginellini) with description of a new species

**DOI:** 10.3897/zookeys.183.2229

**Published:** 2012-04-19

**Authors:** Xia Gao, Min Huang, Yalin Zhang

**Affiliations:** 1 Key Laboratory of Plant Protection Resources and Pest Management, Ministry of Education, Entomological Museum (P.O. Box 55#), College of Plant Protection, Northwest A & F University, Yangling, Shaanxi 712100, Chinas; 2 Hanzhong Agricultural Technology Extension Center, Hanzhong, Shaanxi 723000, China

**Keywords:** Homoptera, Auchenorrhyncha, taxonomy

## Abstract

The two leafhopper species in the genus *Parazyginella* Chou & Zhang from China are reviewed and illustrated including one new species, *Parazyginella tiani***sp. n.** whichis described. A key to separate males of the two species is provided.

## Introduction

Zyginellini is one of the smaller tribes in the large leafhopper subfamily Typhlocybinae. Members of the subfamily feed on trees, shrubs and herbs and some occur on economic crops including two species of Zyginellini, *Zyginella mali* (Yang) and *Zyginella minuta* (Yang), which damage apple trees in China. Members of this tribe can be distinguished by their usual bright coloration with distinct patterns and by the hindwing venation with the vannal veins separate apically, with only one transverse vein visible and submarginal vein extended directly to vein CuA, forming one open cell (Fig. 13).

**Figures 1–4.**
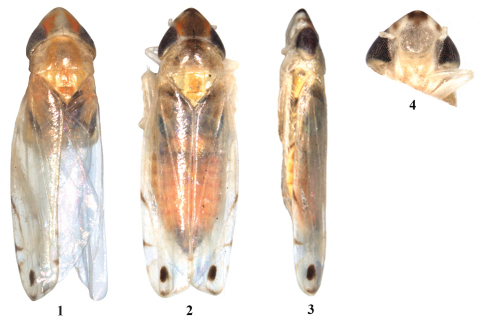
**1** *Parazyginella lingtianensis*, dorsal habitus **2–4** *Parazyginella tiani* sp. n. **2** dorsal habitus **3** lateral habitus **4** face.

The Oriental Zyginellini genus *Parazyginella* was erected by Chou and Zhang (1985) with *Parazyginella lingtianensis* from Guangxi, China as its type species. There have been no further reports of this genus which is recognized by its depressed body form and a dark spot near the apex of the forewing (Figs 1–3). In this paper, we describe a second species, *Parazyginella tiani* sp. n.from Yunnan, China. The type specimens of the new species are deposited in the collections of the Entomological Museum, Northwest A & F University, Yangling, China (NWAFU) and The Natural History Museum, London (BMNH).

## Systematics

### Parazyginella

Chou & Zhang, 1985

http://species-id.net/wiki/Parazyginella

Parazyginella Chou & Zhang, 1985: 295; Zhang 1990: 174.

#### Type species.

*Parazyginella lingtianensis* Chou & Zhang, 1985

#### Description.

Body flattened. Vertex conically produced, middle length nearly equal to width and length of pronotum; coronal suture distinct. Head and pronotum whitish yellow. Scutellum and venter yellow. Forewing with base of 1^st^ and 4^th^ apical cells at same level; 3^r^^d^ apical cell triangular and with a dark spot; hind margin of brochosome field and veins in apical area of wing touched with dark brown.

Abdominal apodemes elongate, reaching beyond 5th abdominal sternite.

Male pygofer strongly sclerotized, with short, finger-like process caudo-dorsally and few scattered microsetae. Subgenital plates broad at base, distally abruptly tapered to short narrow apex, with few macrosetae basally. Paramere simple, with central part expanded, apical part tapering to acute apex and strongly bent. Connective with short arms and medial lobe present. Aedeagus asymmetrical with basal part strongly sclerotized with short preatrium and large dorsal apodeme, the latter laterally compressed with distal anterior region membranous and distal posterior region strongly curved anteriorly; shaft elongate, with a single elongate apical process on one side; gonopore obscure.

*Parazyginella* resembles *Zyginella* Löw, but differs in its more greatly developed dorsal apodeme of the aedeagus and male pygofer with a dorsal finger-like process and without long macrosetae (Figs 16, 17).

#### Distribution.

China(Guangxi, Yunnan).

#### Key to species of *Parazyginella*

**Table:** 

1	Head without brown markings. Aedeagal shaft in lateral view similar in width throughout length, process moderately long and slim, lying parallel to shaft (Figs 10, 11)	*Parazyginella lingtianensis*
–	Head with brown markings (Figs 2–4). Aedeagal shaft in lateral view slightly expanded from midlength to near apex, process long and stout, directed slightly towards basal apodeme (Figs 21, 22)	*Parazyginella tiani* sp. n.

### Parazyginella lingtianensis

Chou & Zhang, 1985

http://species-id.net/wiki/Parazyginella_lingtianensis

Figs 1 5–12

Parazyginella lingtianensis Chou & Zhang, 1985: 295; Zhang 1990: 174

#### Description.

**Figures 5–12.**
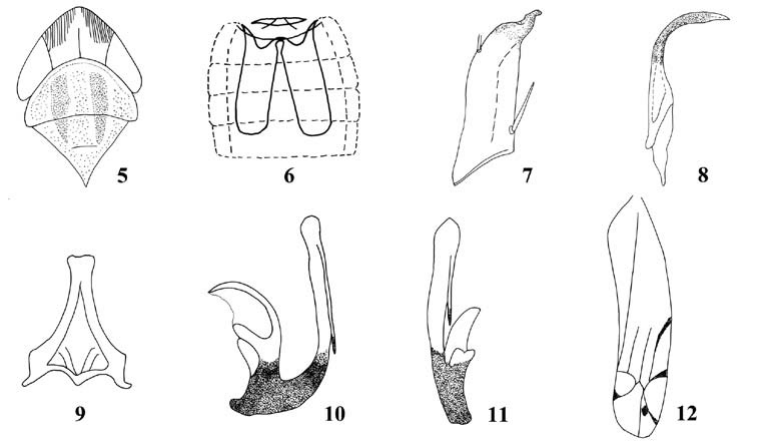
*Parazyginella lingtianensis* (after Chou and Zhang 1985) **5** Head, dorsal view **6** Abdominal apodeme **7** Subgenital plate **8** Paramere **9** Connective **10** Aedeagus, lateral view **11** Aedeagus, posterior view **12** Forewing.

Head and pronotum whitish yellow. Scutellum and venter yellow; vertex and pronotum either side of midline marked with orange (Figs 1, 5); 3^rd^ apical cell of forewing with dark elliptical spot (Figs 1, 12).

Abdominal apodemes reaching middle part of 6th abdominal sternite (Fig. 6).

Subgenital plates with one macroseta near base, apex beak-like (Fig. 7). Paramere slightly expanded subapically (Fig. 8). Aedeagal shaft in lateral view similar in width throughout length, process moderately long and slim, lying parallel to shaft (Figs 10, 11).

#### Body length.

Male 3.00 mm (including wing).

#### Material examined.

*Holotype*, male, China: Guangxi Prov., Lingchuan, Lingtian, 5 June 1984, coll. Lu Xiaolin, lamp (NWAFU).

#### Distribution.

China (Guangxi).

#### Remarks.

The male genitalia of the unique type could not be found. Therefore the pygofer (originally not figured) could not be examined and compared to the new species. Also, the original figure of the aedeagus (shown here, Fig. 10) did not show the membranous area of the basal apodeme shown in our new species. We conclude that this area was probably overlooked and add a line to the figure to show its approximate position.

### Parazyginella tiani sp. n.

urn:lsid:zoobank.org:act:A19A9268-1164-43C8-8B94-0C0CE69CBA7F

http://species-id.net/wiki/Parazyginella_tiani

Figs 2–4 13–22

#### Description.

Head and pronotum whitish yellow; scutellum and venter yellow; vertex with disc and apex dark brown, orange laterally; face sordid white, with a dark brown patch above antennae; dorsum of abdomen stramineus. Forewing with dark spot in 3^rd^ apical cell (Figs 2, 3).

Abdominal apodemes nearly reaching end of 6th abdominal sternite (Fig. 14).

Male pygofer with short, sclerotized, sickle-like process caudo-dorsally (Figs 16, 17). Subgenital plates with two macrosetae near base, apex digitate with few microsetae (Figs 18, 20). Paramere subapically with row of fine setae on outer margin and row of sensory pits on inner margin; with curved apical part with sinuate ridge (Figs 18, 19). Aedeagal shaft in lateral view slightly expanded from midlength to near apex, process long and stout, directed slightly towards basal apodeme, apex ornamented (Figs 21, 22).

#### Body length.

2.95–2.98 mm (including wing).

#### Material examined.

*Holotype*, male, China: Yunnan Province, Sanchahe, 7 June 1991, coll. Tian Rungang (NWAFU). *Paratypes*, two males, seven females, same data as holotype (NWAFU, BMNH).

#### Distribution.

China (Yunnan).

#### Remarks.

The new species resembles *Parazyginella lingtianensis* but differs in having brown markings on the vertex (compare Figs 1 and 2) and different shaped aedeagus as noted in the key.

#### Etymology.

The new species is named after the collector’s family name in gratitude.

**Figures 13–22.**
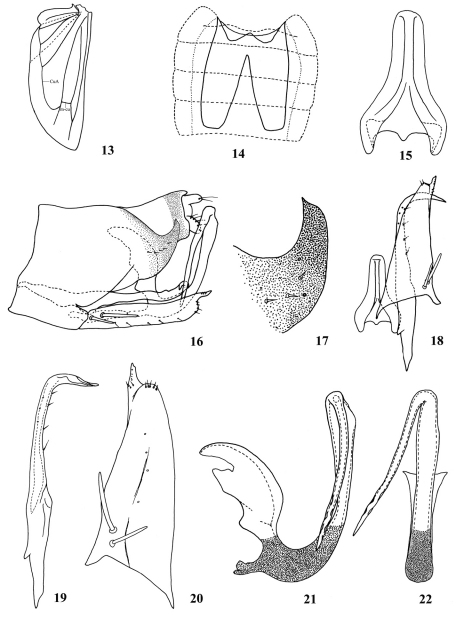
*Parazyginella tiani* sp. n. **13** Hindwing **14** Abdominal apodeme **15** Connective **16** Genital capsule, lateral view **17** Apical part of male pygofer **18** Paramere, connective, subgenital plate, ventral view **19** Paramere **20** Subgenital plate **21** Aedeagus, lateral view **22** Aedeagus, posterior view.

## Supplementary Material

XML Treatment for Parazyginella

XML Treatment for Parazyginella lingtianensis

XML Treatment for Parazyginella tiani
